# Current Status and Future Prospects in Herbicide Discovery

**DOI:** 10.3390/plants8090341

**Published:** 2019-09-11

**Authors:** Franck E. Dayan

**Affiliations:** Department of Bioagricultural Sciences and Pest Management, 1177 Campus Delivery, Colorado State University, Fort Collins, CO 80523, USA; franck.dayan@colostate.edu; Tel.: +1-662-816-6214

**Keywords:** amino acid biosynthesis, lipid biosynthesis, mechanism of action, plastoquinone biosynthesis, pyrimidine biosynthesis, target site

## Abstract

Herbicides represent about 60% of the pesticides (by volume) used worldwide. The success of herbicides can be attributed in part to a relatively steady discovery of one unique mechanisms of action (MOA) every two years from the early 1950s to the mid-1980s. While this situation changed dramatically after the introduction of glyphosate-resistant crops, evolution of resistance to glyphosate has renewed the agrichemical industry interest in new chemistry interacting with novel target sites. This review analyses recent characterization of new herbicide target sites, the chemical classes developed to inhibit these target sites, and where appropriate the innovative technologies used in these discovery programs.

## 1. Introduction

The first synthetic herbicide was discovered in the early 1940s [1] and its efficacy and selectivity caused a paradigm change in agricultural weed management practices. New herbicide mechanisms of action (MOA) were discovered at a relatively steady rate of one unique MOA every two years from the early 1950s to the mid-1980s. Today, herbicides represent about 60% of the pesticides used worldwide, and most large-scale crop production systems rely extensively on synthetic herbicides to manage weeds. This has led to the relatively slow but steady evolution of many herbicide-resistant (HR) biotypes. The introduction of glyphosate-resistant (GR) crops in the last 25 years has compounded the selection pressure imposed by the repeated application of herbicides over larger areas. Managing these HR plants is problematic and the lack of control threatens farm profitability while challenging environmentally beneficial farming practices (e.g., no-till) [2,3]. The emergence and spread of HR weeds will require farmer cooperation to successfully control them [4].

While a tremendous commercial success, GR crops have been detrimental to herbicide discovery programs, causing a lapse in innovative research and development programs and a dearth of new chemistry with novel mechanisms of action (MOA) [5,6]. Factors that have contributed to a reduced investment in herbicide research and development are multifold and have been discussed elsewhere but include [7]:

- The success of GR crops that revolutionized weed management [8].
- The increased cost of R&D programs for production of a single new active ingredient from $184 million in 2000 to nearly $286 million in 2016 [9].
- The increased barriers imposed by toxicological and environmental regulations that must be fulfilled to ensure safety of the products [10].
- The severe attrition of the Agchem industry from more than 100 R&D companies to a few dominating companies [11]. This may in part be due to late stage failures (duPont), expense of liabilities and the depth of intellectual property.

Readers are encouraged to read Gerwick’s review on the problems facing the agchem industry and Duke’s review on why no new herbicide modes of action have been commercialized in recent years to have a broader analysis of these problems [6,12].

So what are the current status and future prospects in herbicide discovery? Herbicides are small (usually <500 MW) molecules that tend to target plant-specific processes. Generally speaking, herbicides can be grouped into three main categories: 1) herbicides that target biochemical pathways and physiological processes involved with photosynthesis, 2) herbicides that inhibit the formation of biological building blocks (i.e., sugars, amino acids and fatty acids) or their assembly into macromolecules, and 3) herbicides with other modes of action (Table 1). The many different active ingredients can be categorized based on their physicochemical properties [13] or organized around their respective mechanisms of action (Table 1). This low number of mechanisms of action is somewhat surprising, considering the thousands of potential molecular target sites that exist in plants and the hundreds of thousands of molecules screened for herbicidal activity every year. 

This review will not cover the known mechanisms of action, and readers interested in the topic are referred to the original reports and several reviews on that topic for more information (e.g., [14,15]). However, a good knowledge of herbicide target sites and their mechanisms of action is crucial to decipher the way new herbicides may exert their action.

As mentioned above, no herbicides with truly new molecular targets had been introduced in the past 30 years. Yet, the need for new tools is more dire than ever, especially to combat HR weeds, and in particular those that have evolved resistance to glyphosate [16]. Though there does not appear to be a ‘silver bullet’ coming down the Agchem pipeline, there has been a recent flurry of reports of new mechanisms of action. So what has changed?

The dominance of glyphosate has been a destabilizing force affecting other agchem companies’ decisions to move forward with new chemistry discovered through their own R&D programs. As mentioned before, the potential market shares of new compounds were not sufficient to justify the cost of developing these products. While the Agchem market is still relying on glyphosate for weed control in all the major row crops, the emergence of GR weeds has begun to impact the current usefulness and future prospect of glyphosate. Indeed, farmers are already returning to older chemistry to control GR weeds. In this new environment, companies may be projecting that the time is ripe for introducing new chemistry and new MOA. This seems to be reflected with recent presentations at the 2019 International Union of Pure and Applied Chemistry (IUPAC) congress on plant protection in Ghent, Belgium [17] and the Agrochemical Division of American Chemical Society’s program at their 2019 national meeting in San Diego, CA. Additionally, several new startup companies have developed innovative technologies to explore new chemical spaces and/or facilitate the elucidation of their target sites. For example, MoA technology uses in vivo high throughput platforms, proprietary bioinformatics and Artificial Intelligence (AI) tools to discover novel herbicides with novel MOAs. Enko Chem Inc. aims to become a leading innovator in crop protection chemistry by utilizing a target-based discovery platform to produce high quality and novel small molecule starting points and a suite of tools and approaches to develop these into product candidates, and Agrematch develops AI and big-data tools for rational identification of molecules with desired biological activity and high potential to become crop protection products while significantly reducing R&D costs and accelerating time to market.

## 2. Novel Mechanisms of Action

### 2.1. Lipid Biosynthesis

Lipid synthesis involves several biochemical pathways leading to the formation of important building blocks for membranes, cuticles and waxes necessary for plant survival. Consequently, it has been the target of several herbicide classes. 

**Figure 1:**
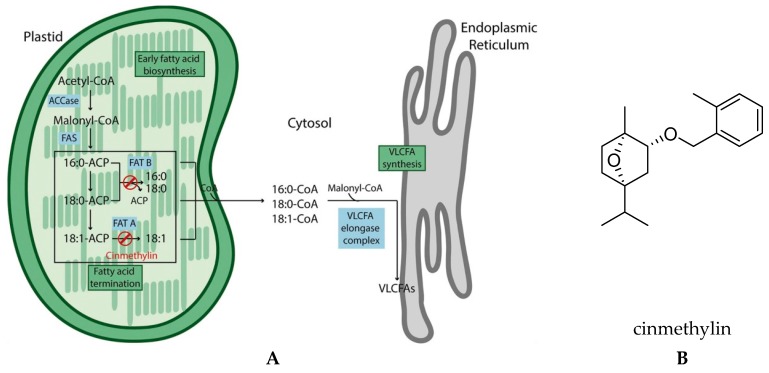
(**A**) Overview of fatty acid biosynthesis and herbicide targets in plant cells. Inhibition of acetyl-CoA carboxylase (ACCase) by haloxyfopmethyl or tepraloxydim disrupts early fatty acid biosynthesis. Cinmethylin prevents the release of both unsaturated and saturated fatty acids from the plastids through inhibition of fatty acid thioesterase (FAT) A and B, respectively. Inhibitors of very-long-chain fatty acid (VLCFA) biosynthesis act at the endoplasmic reticulum. ACP: acyl carrier protein; CoA: Coenzyme A. From [18] with permission. (**B**) Structure of cinmethylin.

Acetyl-coenzyme A carboxylase (ACCase) catalyzes the first committed step to fatty acid synthesis (Figure 1A). Cyclohexanediones (e.g., sethoxydim) and aryloxyphenoxypropionates (e.g., diclofop-methyl) are the two major groups of herbicides targeting this enzyme. Inhibitors of ACCase are grass-selective because two forms of ACCase occur in plants. A prokaryotic form is insensitive to these herbicides and is found only in the plastids of dicotyledonous plants, whereas the herbicide-sensitive eukaryotic form is found in the cytoplasm of all plants and in the plastids of grasses. Non-grass plants are resistant because they can sustain lipid biosynthesis in the presence of such herbicides. 

While there are a host of enzymes catalyzing the many subsequent steps in fatty acid synthesis, the only other herbicide target site in this pathway are the very long chain fatty acid elongases (VLCFAE) (Figure 1A). These enzymes are much further down the metabolic pathway, responsible for synthesis of waxes, cutins, and suberins. VLFCAEs are the targets of several classes of herbicides, including the chloroacetanilides (e.g., alachlor), the thiocarbamates (e.g., EPTC), and the oxyacetamides (e.g., flufenacet). Inhibition of VLFCAEs by these herbicides results in decreased growth and leaf curling or twisting [14].

#### Fatty Acid Thioesterase (FAT)

An exciting new development has been the discovery of new target sites in fatty acid synthesis of an old herbicide, cinmethylin (Figure 1B). These are the fatty acid thioesterases (FAT) (Figure 1B) [18]. FATs are plastid localized enzymes that mediate the release of fatty acids from its acyl carrier protein (ACP) which is necessary for FA export out of the chloroplast and transfer to the endoplasmic reticulum as fatty acyl-CoAs. 

FATs are inhibited by cinmethylin, a natural product-like benzyl ether derivative of 1,4-cineole that was first described by Shell in 1981 and commercialized in 1989 [19]. Plants treated with cinmethylin have reduced levels of the saturated C14:0 and C16:0 fatty acids, indicating that the herbicide inhibits both classes of FAT proteins [18]. The direct interaction of cinmethylin with FAT proteins was confirmed by fluorescence-based thermal shift assays and co-crystallization of cinmethylin within the FAT enzyme.

### 2.2. Plastoquinone Biosynthesis

**Figure 2:**
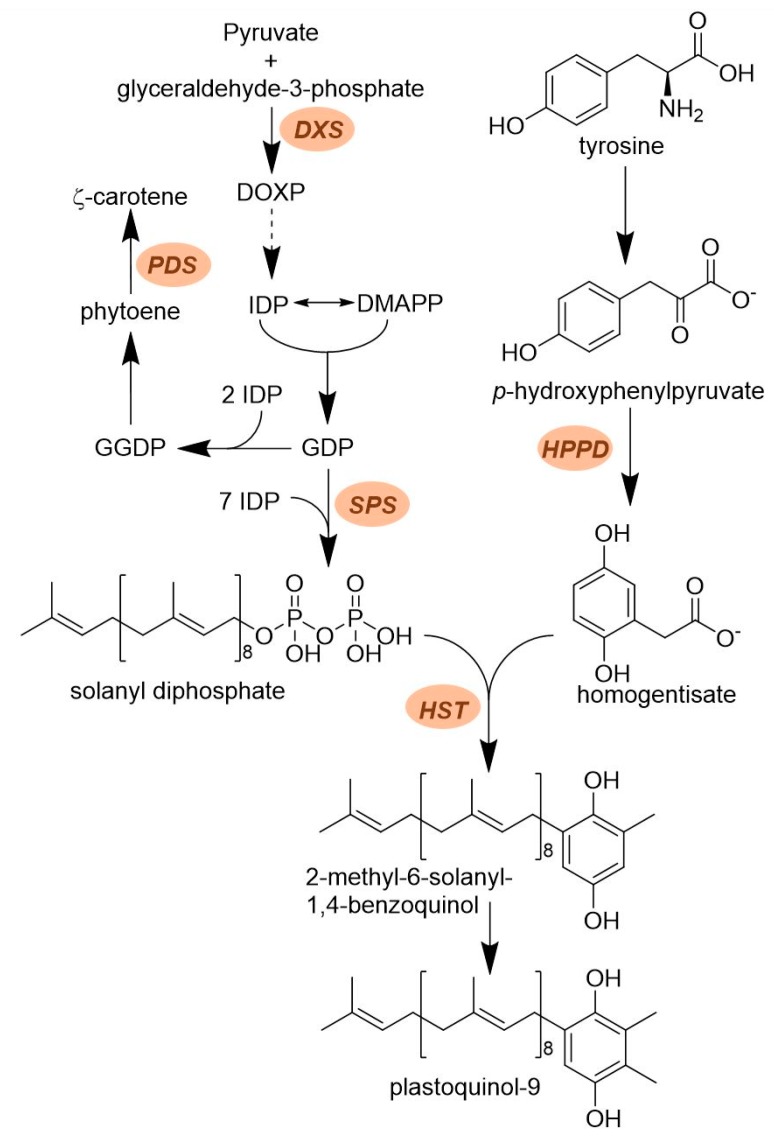
Overview of the relationship between carotenoid and prenyl quinone biosynthesis. Biosynthesis of carotenoids and plastoquinone requires the MEP, terpenoid and homogentisate pathways. Older chemistry such as clomazone inhibits 1-deoxy-D-xylulose 5-phosphate synthase (DXS), the first step in the MEP pathway; a number of chemical classes inhibit carotenoid biosynthesis by targeting phytoene desaturase (PDS); the newer triketone herbicides inhibit p-hydroxyphenylpyruvate dioxygenase (HPPD) involved in homogentisate biosynthesis. The two newest target sites affect solanyl diphosphate synthase (SPS) responsible for the synthesis of the terpenoid tail of plastoquinone or homogentisate solanesyl transferase (HST), the enzyme combining solanyl diphosphate and homogentisate to form a plastoquinone precursor.

Plastoquinone is a lipid (prenyl) quinone with important biological functions. It is best known for its role as an electron acceptor in the light reaction of photosynthesis. Specifically, it accepts electrons from photosystem II and transfers them to the cytochrome b6 complex. Its importance in agrochemistry cannot be overstated. Some of the earliest commercial herbicides (e.g., triazines, ureas, and nitriles) inhibit photosynthesis by competing for the plastoquinone binding site on PSII. Later on, interest in plastoquinone renewed with the discovery of triketone herbicides that inhibit *p*-hydroxyphenylpyruvate dioxygenase (HPPD), a key enzyme in plastoquinone synthesis (Figure 2). These herbicides cause bleaching of photosynthetic tissues because plastoquinone is required for the activity of phytoene desaturase [20], a well-known target of herbicides inhibiting carotenoid biosynthesis (Figure 2). Consequently, industry started focusing on the biosynthesis pathway of plastoquinone in hope of identifying additional herbicide target sites. Its biosynthesis involves the convergence of two pathways (Figure 2). On one hand, the quinone head is derived from tyrosine and involves HPPD to form homogentisate. On the other hand, the lipophilic tail is derived from the 2-C-Methyl-D-erythritol 4-Phosphate (MEP)-derived terpenoid pathway (Figure 2). 

#### 2.2.1. Solanyl Diphosphate Synthase (SPS)

**Figure 3:**
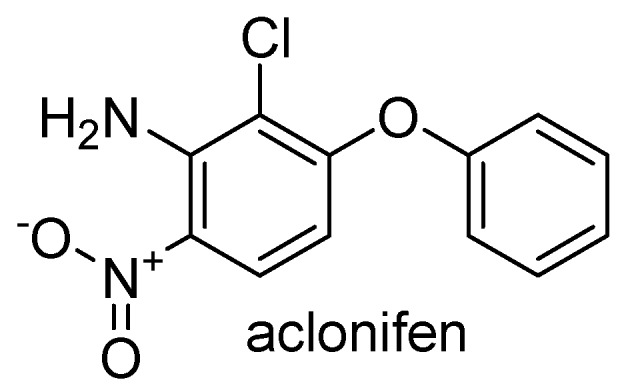
Structure of aclonifen, an inhibitor of chloroplastic solanyl diphosphate synthase (SPS).

The building block of the lipid tail of plastoquinone is solanyl diphosphate. It is obtained by the activity of solanyl diphosphate synthase (SPS) which catalyzes the sequential addition of seven isopentenyl diphosphate to geranyl diphosphate [21]. A collaboration between the herbicide discovery group of Bayer CropScience and Targenomix recently discovered using a systems biology approach that aclonifen (Figure 3) causes bleaching of treated plants by inhibiting SPS. Aclonifen is a relatively old diphenylether herbicide whose MOA was unknown. The binding of aclonifen to SPS was confirmed by crystallography. Phenylalanine residues within the catalytic domain of SPS are involved in the binding of aclonifen. Plants possess three genes encoding SPS. Two of the genes encode for SPS1 and SPS2 proteins that are localized in the chloroplast and involved in plastoquinone synthesis [22]. The other gene encodes for the mitochondrial isoforms (SPS3) involved in ubiquinone synthesis [22]. SPS1 and SPS2 are sensitive to aclonifen, whereas SPS3 is insensitive to the herbicide. 

#### 2.2.2. Homogentisate Solanesyl Transferase (HST)

As mentioned above, plastoquinone biosynthesis involves the convergence of homogentisate and terpernoid synthesis. This step is catalyzed by homogentisate solanesyl transferase (HST) [23]. HST catalyzes the prenylation and decarboxylation of homogentisate to form 2-methyl-6-solanesyl-1,4-benzoquinol, the first intermediate in plastoquinone-9 biosynthesis. (Figure 2). This enzyme was known to be sensitive to inhibition by haloxydine [23]. Haloxydine acts as a suicide inhibitor mimicking homogentisate binding.

**Figure 4:**
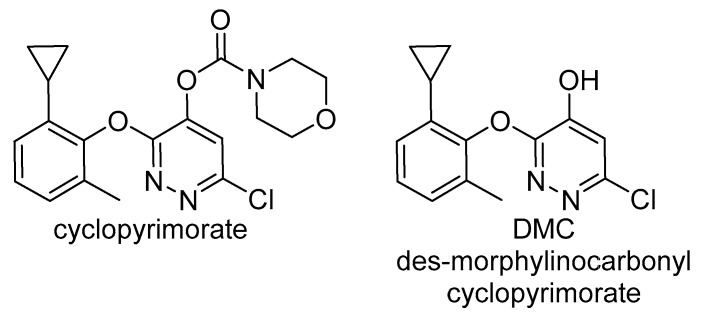
Cyclopyrimorate and its bioactive metabolite des-morpholinocarbonyl cyclopyrimorate (DMC).

Mitsui Chemical Agro Inc. reported the discovery and development of cyclopyrimorate (Figure 4) as a new bleaching herbicide inhibiting HST. This herbicide was discovered in a program aiming at combining the pharmacophore backbone of credazine and pyridafol. Structure optimization against the weeds *Scirpus juncoides* (sedge) and *Sagittaria trifolia* (threeleaf arrowhead) demonstrated that the cyclopropane ring and the methyl group at the ortho positions (2 and 6, respectively) on the phenyl ring were critical for activity. The hydrophobicity of the moiety at position 2 can modulate activity, with cyclopropane being optimal. Additionally, a hydroxy group at position 4 on the pyridazine ring is important. Extensive biochemical work determined that plants treated with this herbicide have decreased levels of chlorophyll, carotenoids and plastoquinone, and accumulate homogentisate. The effect of the herbicide was strongly reversed by decyl plastoquinone and moderately reversed by homogentisate, suggesting that cyclopyrimorate targeted HST. Further work demonstrated that cyclopyrimorate was a proherbicide that needed to be bioactivated into its des-morpholinocarbonyl cyclopyrimorate (DMC) metabolite (Figure 4) to inhibit HST [24]. In planta metabolism of cyclopyrimorate into DMC releasing the free hydroxy group on position 4 of the pyridazine ring is critical for bioactivation of this herbicide. The *I*_50_ values for cyclopyrimorate and DMC on HST were 3.9 and 561 μM, respectively. DMC is a competitive inhibitor of HST for homogentisate but uncompetitive toward the prenyl diphosphate (Figure 2). 

Bleaching symptoms are the results of a dramatic decrease in plastoquinone levels in treated plants. Since this target site is downstream enzyme of HPPD in the plastoquinone biosynthesis pathway, activity of cyclopyrimorate is enhanced in tank mix with 4-HPPD inhibitors. This herbicide will be developed for weed management in rice paddies, including acetolactate synthase (ALS) resistant weed species with projected commercialization in 2020.

### 2.3. Amino Acid Biosynthesis and Protein Regulation

#### 2.3.1. Dihydroxy-Acid Dehydratase (DHAD)

**Figure 5:**
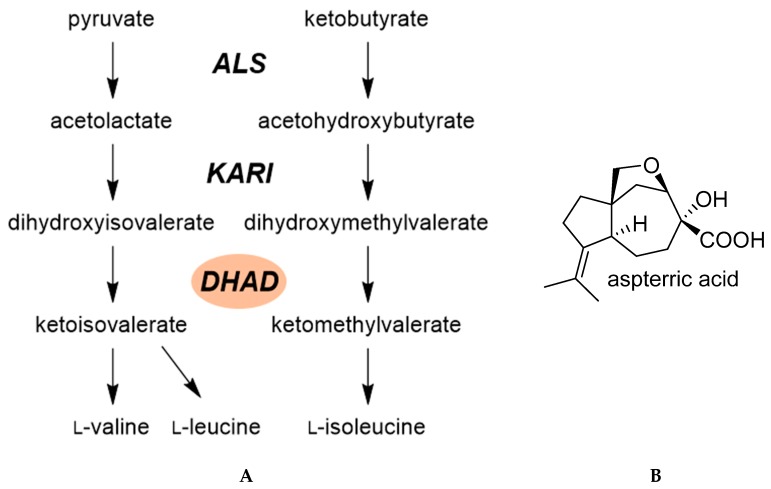
(**A**) Key enzymes involved in branched chain amino acid biosynthesis. DHAD is the most recent putative herbicide target site in this pathway. (**B**) Structure of the microbial metabolite aspterric acid, an inhibitor of DHAD. ALS: acetolactate synthase; KARI: acetohydroxy acid isomeroreductase; DHAD: dihydroxy acid dehydratase.

A large number of herbicides inhibit branched chain amino acid biosynthesis by targeting acetolactate synthase, the first step committed in this pathway (Figure 5) [14]. In light of the commercial success of this chemistry, industry has searched for chemicals that could inhibit this pathway by other means. A number of inhibitors of acetohydroxy acid isomeroreductase have been discovered but none of them have been developed into commercial products. 

An innovative resistant-gene-directed discovery approach led to the discovery of a new herbicide target site in the branched chain amino acid pathway [25]. Aspterric acid is a natural herbicide produced by the soil fungus *Aspergillus terreus* (Figure 5). A research group at University of California Los Angeles analyzed the microbial gene cluster involved in the biosynthesis of this microbial phytotoxin. They discovered that that the cluster also included a paralog form of dihydroxy acid dehydratase (DHAD), the last common enzyme of the branched chain amino acid biosynthesis pathway (Figure 5). Further work demonstrated that aspterric acid targets DHAD, and the DHAD paralog present in the gene cluster was aspterric acid-resistant, its target enzyme [25]. Aspterric acid is a relatively weak phytotoxin which may not rise to a successful commercial herbicide, but it might serve as a structural backbone to elaborate new herbicide classes with improved physicochemical properties. As well, it is not clear whether DHAD is a good target site for herbicide to control weeds under agronomic conditions, and more work must be carried out to validate it as a desirable target site. Nevertheless, one of the advantages of this microbial gene co-clustering analysis is that it can lead to the discovery of genes involved in a phytotoxin biosynthesis, the identification of its target site, and the isolation of a herbicide-resistant form of this target site [26].

#### 2.3.2. 3-Dehydroquinate Synthase

**Figure 6:**
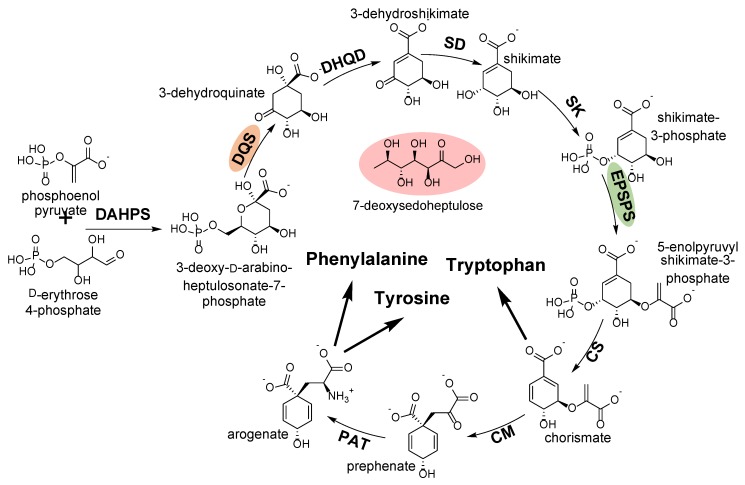
Shikimate pathway and aromatic amino acid biosynthesis showing the metabolites formed at each step catalyzed by the enzymes abbreviated in bold. The target site of glyphosate is EPSPS (green). The target site of the antimetabolite 7-deoxysedoheptulose (red) is DQS (orange). DAHPS: 3-deoxy-d-arabino-heptulosonate-7-phosphate synthase, DQS: 3-dehydroquinate synthase, DHQD/SD: 3-dehydroquinatede hydratase, SK: shikimate kinase, EPSPS: 5-enolpyruvylshikimate 3-phosphate synthase, CS: chorismate synthase, CM: chorismate mutase, PAT: prephenate aminotransferase.

The shikimate pathway is one of the central pathways associated in plant metabolism, providing the carbon skeletons for the aromatic amino acids l-tryptophan, l-phenylalanine, and l-tyrosine (Figure 6) and many important secondary metabolites (e.g., chlorogenic acid, alkaloids, glucosinolates, auxin, tannins, suberin, lignin and lignan, and tocopherols) [27]. It is estimated that at least 30% of all fixed carbon is directed through this pathway to support the flux required to produce these plant components.

Since mammals cannot synthesize these amino acids, this pathway is particularly desirable as a potential target for herbicides. To date, glyphosate is the only herbicide targeting this pathway by acting as an irreversible inhibitor of 5-enolpyruvylshikimate-3-phospate synthase (Figure 6). While glyphosate slowly depletes the pools of aromatic amino acids, its herbicidal activity is associated with a deregulation of the shikimate pathway, leading to accumulation of high levels of shikimate-3-phosphate and shikimate and siphoning of carbon and phosphate from other pathways, disrupting more than just the shikimate pathway [14].

Recently, a group from Tübingen University (Germany) has focused on antimetabolites as novel structural backbones to discover inhibitors affecting new target sites. Antimetabolites are interesting molecules as they inhibit enzymes by mimicking their physiological substrates. Their study identified the rare sugar 7-deoxy-sedoheptulose (7dSh) as an inhibitor of 3-dehydroquinate synthase (Figure 6), a key enzyme of the shikimate pathway. 7dSh is active at low micromolar range [28]. The growth of plants treated with 25 μM 7dSH was inhibited to the same degree as an equivalent amount of glyphosate. However, treatment with 7dSH caused an accumulation of 3-deoxy-d-arabino-heptulosonate-7-phosphate, the substrate of 3-dehydroquinate synthase (Figure 6), whereas glyphosate caused a rapid accumulation of shikimate, a well-known biomarker of 5-enolpyruvylshikimate 3-phosphate synthase inhibition [29]. Not surprisingly, plants treated with 7dSh have lower levels of free aromatic amino acids (tyrosine, phenylalanine, and tryptophan). This apparently deregulates the biosynthesis of other amino acids, resulting in accumulation of the branched chain amino acids (valine, leucine, and isoleucine), as well as arginine. 7dSh did not have any preemergence activity. However, it controlled velvetleaf (*Abutilon theophrasti*) when applied as a postemergence herbicide at a rate of 2 kg ha^-1^. The addition of an adjuvant was required to obtain this level of activity. On the other hand, 7dSh had no activity on green foxtail (*Setaria viridis*) suggesting selectivity for broadleaf weed control.

#### 2.3.3. Serine/Threonine Protein Phosphatases (PPs)

More than 70% of all proteins have multiple phosphorylation sites and many of these proteins’ activities are regulated via phosphorylation. This is achieved by the concerted action of protein kinases and phosphatases, that account for between 2–4% of the protein-encoding genes of most plants [30,31]. The specificity of protein kinases is based on primary sequence recognition, whereas protein phosphatases tend to be non-discriminate. However, studies across many eukaryote systems confirmed that the phosphatases are not involved in generic dephosphorylation but are in fact as highly regulated as their kinase counterparts. Phosphoprotein phosphatases represent a large group of proteins, that include a sub-class called serine/threonine phosphatases (PPs) [32].

As their names imply, protein serine/threonine phosphatases (PPs) remove phosphate groups bound to serine and threonine residues. PPs are categorized into three subclasses—phosphoprotein phosphatases, metal-dependent protein phosphatases, and aspartate-based phosphatases. The PPs in plants belongs to the phosphoprotein phosphatase sub-class [32]. 

**Figure 7:**
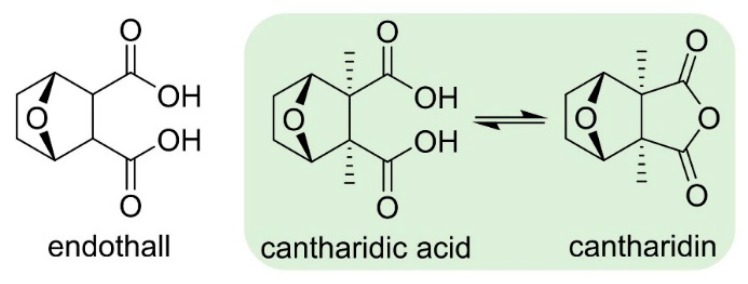
Structure of endothall, cantharidin and its dicarboxylic acid analog.

PPs are the target of endothall (Figure 7), an old herbicide that was first commercialized in the 1950s. Endothall induces severe growth inhibition [15]. Endothall is a structural analog of cantharidin, a natural product from the blister beetle (*Epicauta* spp.) and the Spanish fly (*Lytta vesicatoria*) (Figure 7). Both of these molecules cause similar symptoms on plants [33]. 

Endothall and cantharidin both inhibit plant serine/threonine protein phosphatases in a time-dependent manner, suggesting that these compounds act as slow, irreversible inactivators of the serine/threonine protein phosphatase activities [33]. The catalytic domain of all PP highly conserved across animals, plants and protozoans. Inhibitors, such as cantharidin and endothall, bind to a hydrophobic pocket of the PP active site. Endothall is a very effective herbicide to manage weeds in aquatic environments [34]. 

### 2.4. Pyruvate Dehydrogenase Complex (PDHc)

Pyruvate dehydrogenase complex (PDHc) catalyzes the oxidative decarboxylation of pyruvate to form acetate and its subsequent acetylation of coenzyme A (CoA) to produce acetyl-CoA [35]. As such it is a critically important for cellular processes. The complex consists of three enzymes and a number of cofactors. Pyruvate dehydrogenase E1 is a thiamine diphosphate- and Mg^2+^-dependent enzyme catalyzing the first step of the multistep process associated with PDHc [35]. 

**Figure 8:**
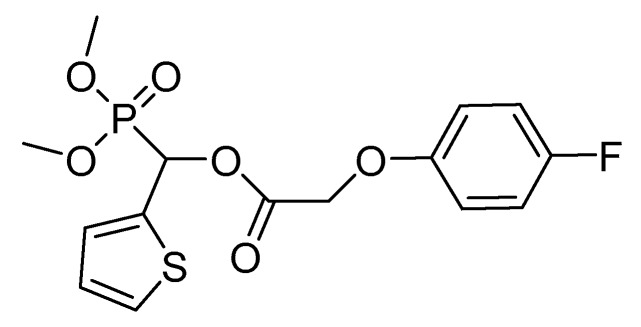
Structure of a potent 1-(substituted phenoxyacetoxy)alkylphosphonate that targets pyruvate dehydrogenase complex (PDHc).

The Institute of Pesticide and Organic Chemistry of Central China Normal University recently reported novel cyclic methylphosphonates (Figure 8) that target pyruvate dehydrogenase complex (PDHc) using molecular docking and three-dimensional quantitative structure-activity relationship studies [36]. Early acetylphosphinates and acetylphosphonates analogs had relatively low herbicidal activity, but these structures served as the basis for structural optimization to generate 1-(substituted phenoxyacetoxy)alkylphosphonate derivatives with notably higher herbicidal activities (Figure 8) [36]. Herbicidal activity is proportional to inhibition of PDHc E1. 

Recent development reported that these PDHc inhibitors were most effective against broadleaf weeds and active at rates ranging from 50 and 300 ai g/ha, whereas they had no effect on maize and rice even at 900−1200 ai g/ha. Some of these compounds also had activity on sedge weeds when applied at 225−375 ai g/ha [37].

### 2.5. Imadazoleglycerol Phosphate Dehydratase (IGPD)

**Figure 9:**
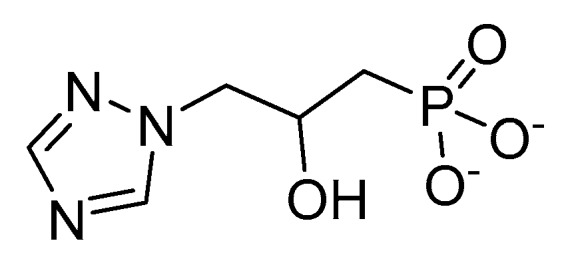
Structure of 2-hydroxy-3-(1,2,4-triazol-1-yl) propylphosphonate, an herbicidal inhibitor of imadazoleglycerol phosphate dehydratase (IGPD).

Imadazoleglycerol phosphate dehydratase (IGPD) catalyzes an important step in histidine biosynthesis in plants and microorganisms. It has been studied for many years as a potential target for herbicides, since this enzyme does not exist in animals. A class of phloem-mobile herbicides (the triazole-phosphonates) act as potent inhibitors of IGPD [38]. Syngenta has been working on this target site for many years. The triazole phosphonate inhibitor 2-hydroxy-3-(1,2,4-triazol-1-yl) propylphosphonate (Figure 9) is structurally similar to the proposed diazafulvene intermediate in IGPD catalysis [39]. Several triazole phosphonate inhibitors have activities similar to glyphosate [40]

Structurally, triazole phosphonate inhibitors consists of three parts, the triazole head, an hydroxylated linker and a phosphate mimick. The position of the hydroxy group alters the binding of the molecules to the catalytic domain of IGPD forming either 5- or 6-membered ring chelates with one of the Mn atom [41].

### 2.6. Dihydroorotate Dehydrogenase (DHODH)

De novo pyrimidine nucleotide biosynthesis (also known as the orotate pathway) consists of six enzymatic steps leading to the formation of uridine monophosphate from carbamoylphosphate, aspartate, and 5-phosphoribosyl-1-pyrophosphate. Because of the central role of nucleotides, inhibition of this pathway is lethal to most organisms. The fourth step is catalyzed by dihydroorotate dehydrogenase (DHODH), which carries out the ubiquinone-mediated oxidation of dihydroorotate to orotate [42].

**Figure 10:**
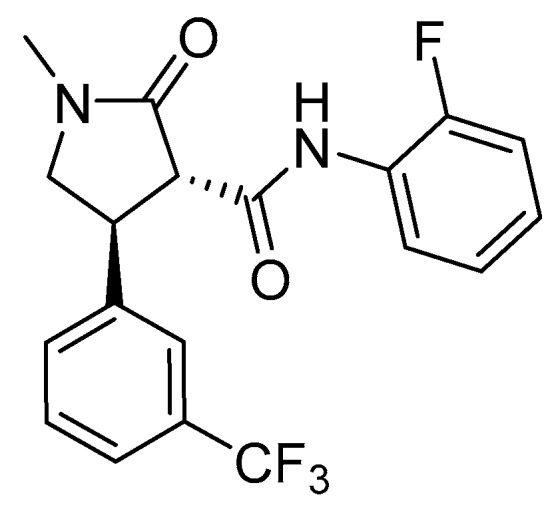
Structure of tetflupyrolimet, an aryl pyrrolidinone anilide targeting Dihydroorotate dehydrogenase, a key enzyme in pyrimidine biosynthesis.

All plant DHODHs are flavoproteins located on the outer surface of the inner mitochondrial membrane. Plant DHODHs have different substrate specificity and inhibition from the animal form of this enzyme [43]. FMC Agricultural Solutions recently announced a new herbicide chemical class (aryl pyrrolidinone anilide) targeting DHODH. The common chemical name of the flagship molecule currently being developed was provisionally approved as tetflupyrolimet (Figure 10). This potent herbicide is selective for grass control in rice. Sensitive plants treated with tetflupyrolimet have no chlorosis but develop a unique stunting phenotype suggesting that they are lacking a key molecule for growth (pyrimidine). The target site was discovered using a combination of forward genetic screens and metabolomics approaches and confirmed by determining intrinsic affinities of specific analogs using biochemical methods [17]. Structure-activity studies determined that the 3*S*-4*R* enantiomer is the active form of this aryl pyrrolidinone anilide, and the 3*R*-4*S* enantiomer had no herbicidal activity. Additionally, the presence of the electron withdrawing groups (fluorine) on the two benzyl rings and the alkylation (methyl group) of the γ-lactam heterocycle are required for herbicidal activity. Tetflupyrolimet competes for the quinone binding site on DHODH. The activity of tetflupyrolimet was about 10-fold greater on the foxtail DHODH enzyme (*I*_50_ = 3 nM) compared to rice (*I*_50_ = 33 nM). However, selectivity for rice is much greater than 10-fold, suggesting that differential metabolism may also contribute to tolerance in rice. Additional work demonstrated that tetflupyrolimet was much less active on animal DHODH. Commercialization of this product is projected to be in 2024.

### 2.7. Peptide Deformylase

**Figure 11:**
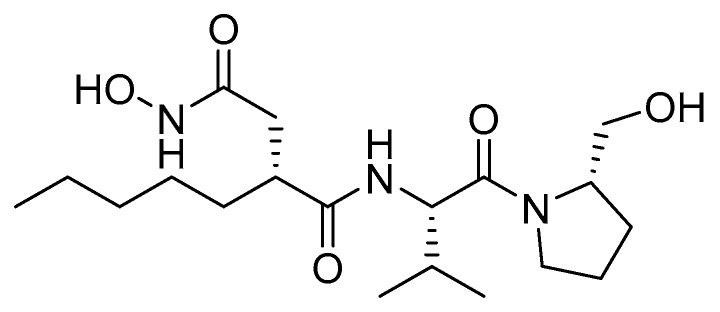
Structure of actinonin, a microbial metabolite targeting chloroplastic peptide deformylase.

In higher plants, synthesis of plastid encoded proteins is initiated with *N*-formylmethionine. Removal of the N-formyl group by a peptide deformylase and the methionine by methionine amino peptidase is necessary to produce the mature protein. The initiator methionine is sometimes retained [44]. Peptide deformylase is the target of actinonin, an hydroxamic acid microbial metabolite produced by soil actinomycetes (Figure 11) [45]. This unique MOA has received a lot of interest and the herbicidal activity of actinonin has been patented, but no commercial product has been developed. Plants treated with actinonin are stunted with bleached foliage which ultimately develop necrotic lesions. It has proved effective on many important weed species [46,47].

### 2.8. DNA Gyrase

**Figure 12:**
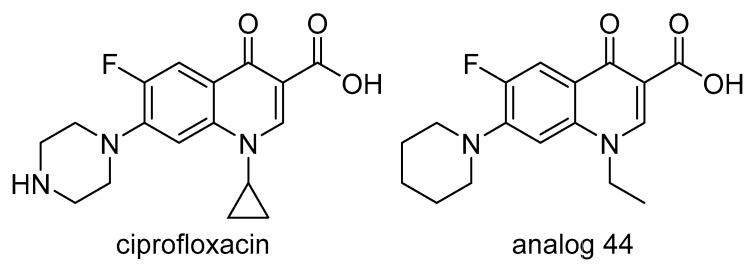
Structure of the fluoroquinolone ciprofloxacin and structure-optimized analog 44 with increased specificity against plants DNA gyrase and better herbicidal profile.

DNA gyrases are prokaryotic Type II topoisomerases that were thought to be absent from most eukaryotes. However, ancestral forms of DNA gyrases may be present in certain organelles of plants and apicomplexans, although their exact functions in replication are not well understood. A research group from the University of Western Australia investigated DNA gyrase as a potential herbicide target site by testing the activity of a number of compounds likely to interact with this enzyme. Several molecules, including the antimicrobial ciprofloxacin (Figure 12), were herbicidal by inhibiting the function of gyrase in higher plants [48]. Three genes (*ATGYRA*, *ATGYRB1*, *ATGYRB2*) encoding for plant gyrases were identified in *Arabidopsis thaliana*. Forward genetic approaches led to the discovery of a point mutation in *ATGYRA* that confers resistance to ciprofloxacin, thus confirming that this gene encodes a functional organelle-localized DNA gyrase that is the target of quinolone antimalarial drugs [49].

Subsequent work exploring the activity of ciprofloxacin analogs on DNA gyrase led to the characterization of the pharmacophore scaffold required for activity and the discovery of structures with improved herbicidal efficacy and diminished antibacterial activity, relative to ciprofloxacin. The optimized experimental analog 44 (Figure 12) had an ethyl side chain and a piperidine ring instead of a cyclopropyl side chain and a piperazine ring attached to the fluoroquinolone scaffold. This molecule was slightly less herbicidal than ciprofloxacin, but its specificity for plant DNA gyrase was superior, leading to a 600-fold increase in selectivity for plants relative to other organisms [50].

### 2.9. Dihydrofolate Reductase (DHFR)

**Figure 13:**
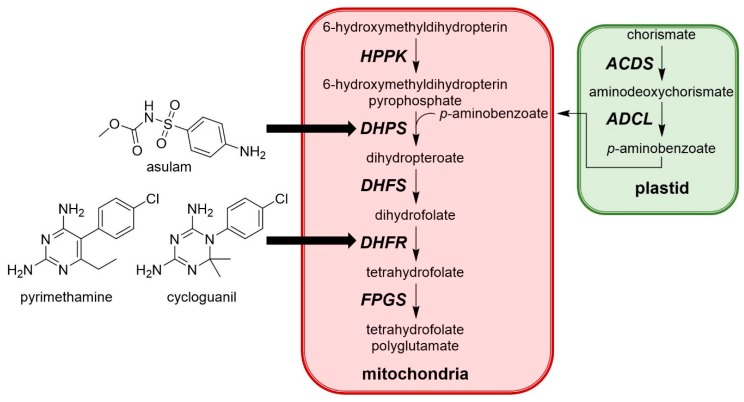
Simplified biosynthesis of folate in higher plants and structures of herbicidal compounds targeting this pathway. HPPK: 6-hydroxymethyldihydropterin pyrophosphokinase; DHPS: dihydropteroate synthase; DHFS: dihydrofolate synthetase; DHFR: dihydrofolate reductase; FPGS: folylpolyglutamate synthetase; ADCS: aminodeoxychorismate synthase; ADCL: aminodeoxychorismate lyase.

The biosynthesis of folate has been the target for pharmaceutical and agrochemical discovery (Figure 13). Folate is an important metabolite required for the synthesis of numerous compounds necessary for plant growth and development. To date, asulam (Figure 13) is only one commercial herbicide to inhibit this pathway by targeting 7,8-dihydropteroate synthetase [51]. This carbamate herbicide is an analogue of 4-aminobenzoate, one of the substrates of 7,8-dihydropteroate synthase, and its selectivity is based on differential metabolic degradation. 

Another enzyme in this pathway, dihydrofolate reductase (DHFR) (Figure 12) has already received some interest as a target for drug development due to its essential role in the synthesis of DNA precursors and some amino acids. A recent study by a group at the University of Western Australia identified DHFR as a potential new target for herbicides based on the herbicidal activity of antimalarial compounds such as pyrimethamine and cycloguanil (Figure 13) [52]. The requirement of two of the three isoforms of DHFR for seed development was identified by knockout mutant analysis. Validation of this enzyme as a new target site was confirmed by screening mutated *Arabidopsis thaliana* seeds for resistance to these antimalarial compounds. A G137D mutation in the isoform 1 of DHFR and a A71V mutation in isoform 2 of DHFR imparted resistance, confirming that the herbicidal activity associated with the antimalarial molecules were due to inhibition of DHFR. This discovery sets the stage for high throughput screening of chemical libraries to identify molecules with better herbicidal profile.

## 3. New Insight on Known Mechanisms of Action

### 3.1. New Insight on Glufosinate Mechanism of Action

**Figure 14:**
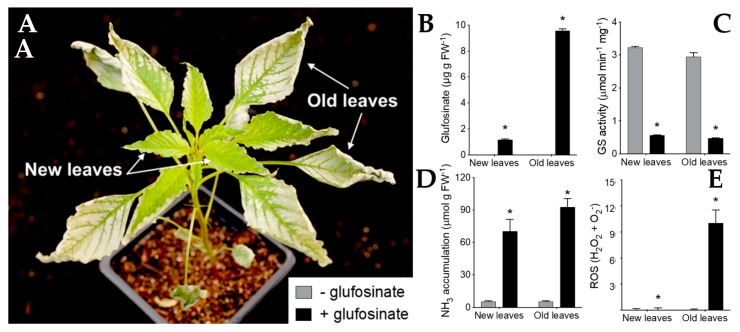
New insight into the mechanisms of action (MOA) of glufosinate. (**A**) Older leaves are more sensitive to glufosinate than meristematic tissue and younger leaves. (**B**) Absorption of glufosinate. (**C**) Inhibition of glutamine synthetase (GS). (**D**) Ammonia accumulation. (**E**) Reactive oxygen species (ROS) accumulation. Reproduced from Takano et al. 2019 with permission.

The MOA of glufosinate has been studied extensively. While the inhibition of glutamine synthetase and subsequent accumulation of ammonia, disruption of amino acid balance, and reduction of both the light and dark reaction of photosynthesis are well documented, these did not account for the rapid desiccation of the foliage induced by glufosinate. New insight on the factors contributing to the contact activity of glufosinate has been reported by the Weed Research Laboratory at Colorado State University. Glufosinate triggers a rapid and massive production of reactive oxygen species (ROS) driving the catastrophic lipid peroxidation of the cell membranes and rapid cell death (Figure 14A) [53]. The effect was proportional to absorption of the herbicide. Interestingly, young leaves were less sensitive to glufosinate. While older leaves absorbed more glufosinate than younger tissues (Figure 14B), similar levels of glutamine synthetase inhibition and ammonia accumulation were observed (Figure 14C,D), indicating that ammonia accumulation was not responsible for the toxicity of this herbicide. In contrast, glufosinate induced a rapid and massive accumulation of ROS in older tissue and almost no ROS in younger tissue (Figure 14E), which correlated directly with the level of injury.

### 3.2. New Insight on Slow-Binding Properties of HPPD Inhibitors

**Figure 15:**
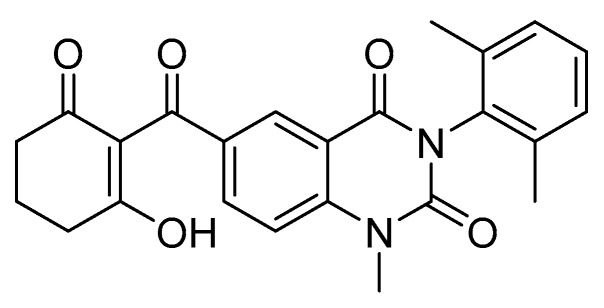
Structure of novel HPPD inhibitor Y13161 (benquitrione).

As mentioned in Section 2.2, inhibition of plastoquinone biosynthesis has been recognized as an excellent target for new herbicide research since the discovery that triketone herbicides inhibit HPPD. These herbicides bind slowly but very tightly (nearly irreversibly) to the catalytic site by coordinating with Fe atom involved in catalysis [54]. A research group based at Central China Normal University provided new molecular insights into the mechanism of 4-hydroxyphenylpyruvate dioxygenase (HPPD) inhibition using enzyme kinetics, X-ray crystallography of *Arabidopsis thaliana* HPPD complexed with herbicides, and computational simulations approaches. This work dissected the interaction between ligand and receptor to discover a novel quinazoline-2.4-dione herbicide benquitrione (Y13161) (Figure 15) [55]. Their analysis suggests that the slow binding properties of HPPD inhibitors may be related to steric hindrance requiring a conformational change on the enzyme upon herbicide binding. Benquitrione has excellent herbicidal activity that compares favorably with that of mesotrione. This molecule also demonstrated selectivity on corn and sorghum, whereas the mesotrione caused injury to sorghum. Finally, the structural features of benquitrione can also serve as a template to develop the next generation of high performance HPPD-inhibiting herbicides.

## 4. Promising New Chemistry

### 4.1. Isoxazolopyridine Herbicides

**Figure 16:**
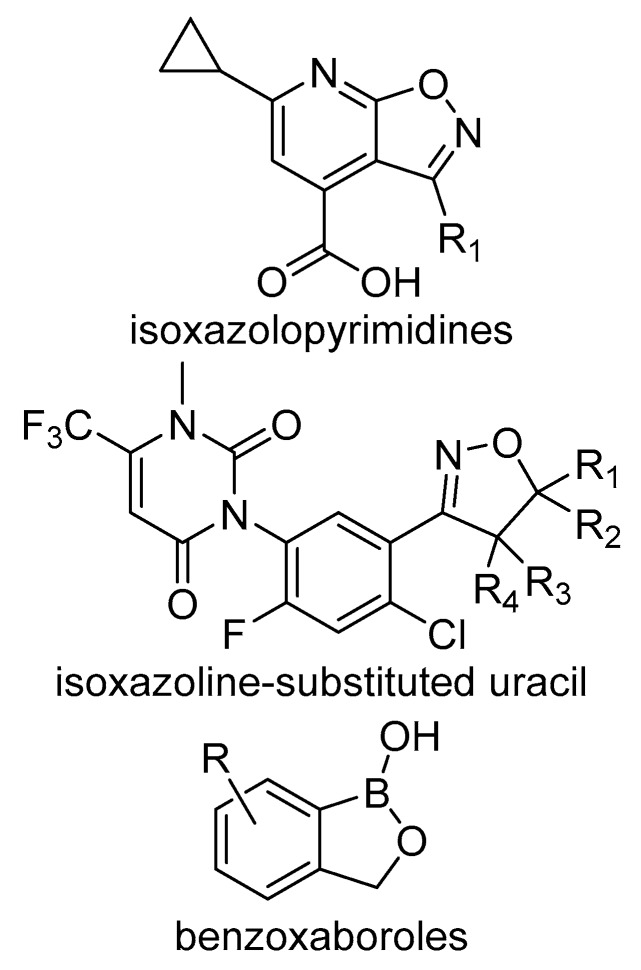
Structural features of the chemical classes mentioned in Section 4.

BASF recently reported a new class of herbicides based on an isoxazolopyridine (OXP) backbone (Figure 16). Some of the compounds have selectivity on monocotyledonous crops while providing excellent post-emergence control of dicot weeds and good activity on some grasses. Sensitive plants treated with OXP herbicides develop necrosis on the foliage and the compounds appear to have systemic activity, with phloem-mobility in dicots but limited translocation in monocots. 

Biochemical/physiological studies excluded known MOA. Therefore, isoxazolopyridines may have a novel mechanism of action on plant photosystems that involves carbohydrate metabolism within the chloroplast. Cellular thermal shift assays suggest that light harvesting protein may be the potential target. Interestingly, a L218V mutation on the D1 protein of photosystem II from metamitron tolerant *Chenopodium album* (Common lambsquarters) provided partial resistance to OXPs, whereas the well-known S264G mutation did not protect plants against the activity of OXPs.

### 4.2. Isoxazoline-Substituted Uracil Herbicides

Sinochem Agrochemicals R&D Co. Ltd. developed novel uracil herbicides containing an isoxazolopyrimidine ring (Figure 16). These compounds effectively control a number of economically important monocot and dicots weeds and appears to be safe to wheat, corn and rice. One of the most promising molecules (SYP-1604) can be used alone or in combination with other herbicides. SYP-1604 spectrum of activity outperforms saflufenacil when applied post and compared favorably to flumioxazin when applied pre. Little is known about its MOA, but it appears to act as an inhibitor of protoporphyrinogen oxidase. 

### 4.3. Benzoxaboroles Herbicides

Benzoxaboroles (Figure 16) are derivatives of boronic acids that were first described over 50 years ago. Interest in these compounds renewed in 2006, after reports of the sugar-binding properties of certain benzoxaboroles were made public. Consequently, most of benzoxaboroles have been described over the last decade [56]. Interest in these structures widened due to their range of biological activities, including the commercialization of two pharmaceutical products. Benzoxaboroles have recently been considered as starting points for new herbicides. Scientists at Corteva explored the chemical space occupied by benzoxaboroles and uncovered a promising area for new herbicide discovery. A limiting factor is the relatively high pka of boronic acid (8.8), but structures with constricted rings, such as the benzoxaboroles, can be designed with lower pka values A number of benzoxaboroles were identified herbicidal hits in initial screens, and plants treated with this type of chemistry developed unique symptoms. However, rates required for activity were high (1–4 kg ha^-1^). Symptoms of plants treated with this class of chemistry varied from stunting to bleaching and necrosis on the leaf margin. While no target site has been identified or reported to date, structure-activity work demonstrated that adding a methyl group enhanced the activity. Replacing this methyl group with cyano group was even more potent. Interestingly, increasing the steric bulk by adding a phenyl ring was advantageous, but decoration with electron withdrawing groups (i.e., chlorophenyl) has a negative impact. Finally, adding alkyl spacer to the phenyl ring positively modulated activity. While no herbicides have arisen from this chemical class, isoxaboroles may lead to new scaffolds to develop new phloem-mobile molecules.

## 5. Conclusions

The time for innovative MOA and chemistry targeting these is overdue. The current crisis experienced by farmers facing difficulties managing weeds that have evolved resistance to many of the existing MOA must be addressed, and new weed management tools are necessary. While the current renewed interest in research and development programs observed in the agrichemical industry, as well as academic and governmental institutions is a positive development, there is no silver bullet chemistry ready to enter the marketplace. Many of the MOA and associated chemistries described in this review are at least 5 years away from commercialization or will fail to reach commercialization due to the many hurdles facing such a process. The aggregation of the agrichemical industry is certain to continue, further limiting diversity in creativity and discovery. One may hope that the few startup companies using truly innovative approaches to herbicide discovery will provide platforms to explore new chemical spaces and biochemical processes. It may also be time to incorporate non-chemical means of weed control, such as mechanical seed destruction (e.g., Harrington seed destructor), robotic weed management, and precision farming.

## Figures and Tables

**Table 1:** Classification of mechanisms of action for current and potential new commercial herbicides discussed in this review.

Group	Type	Mechanism/Target
Biochemical pathways and physiological processes involved with photosynthesis	Light reaction	Photosystem II
		Photosystem I
	Carotenoid	Deoxyxylulose-5-phosphate synthase
		Phytoene desaturase
	Plastoquinone	*p*-Hydroxyphenylpyruvate dioxygenase
		***Homogentisate solanesyltransferase*** ^1^
		***Solanyl diphosphate synthase*** ^1^
	Chlorophylls	Protoporphyrinogen oxidase
	Uncouplers	Oxidative (photo)phosphorylation
Formation of biological building blocks or their assembly into macromolecules	Amino acids	5-enolpyruvylshikimate-3-phosphate (EPSP) Synthase
		Acetolactate synthase
		Glutamine synthetase
		***Dihydroxy-acid dehydratase*** ^1^
	Lipids	Acetyl-CoA carboxylase
		***Fatty acid thioesterase*** ^1^
		Very long chain fatty acid elongases
	Cell walls	Cellulose synthase and others
	Microtubule assembly	α- and/or β-Tubulin
	Microtubule organization	Microtubule organizing centers
	Folates	Dihydropteroate synthetase
		***Dihydrofolate reductase*** ^1^
	Nucleic acids	***DNA gyrase*** ^1^
		***Dihydroorotate dehydrogenase*** ^1^
Other processes	Protein synthesis	***Peptide deformylase***
	Protein regulation	***Serine-threonine protein phosphatases*** ^1^
	Hormone	Synthetic auxins
		Auxin-transport inhibition

^1^ bold and italics indicates recently described or potentially new mechanisms or targets.
